# Supplementary material for: Potential implementation strategies, acceptability, and feasibility of new and repurposed TB vaccines
Source: PLOS Glob Public Health. 2022 May 3;2(5):e0000076. doi: 10.1371/journal.pgph.0000076 (PMC10021736; doi:10.1371/journal.pgph.0000076)
Supplement: S1 Table — (DOCX) [file pgph.0000076.s001.docx]

**S1 Table. Overview of expertise of interviewees.**

| **Expertise** | **South Africa** | **China** |  | **India** | **Total** |
| --- | --- | --- | --- | --- | --- |
| **National** | 6 | 5 |  | 4 | 15 |
| **Regional** | 1 | 1 |  | 2 | 4 |
| **Local** | 1 | 0 |  | 1 | 2 |
| **TB** | 5 | 3 |  | 6 | 14 |
| **Vaccines** | 4 | 3 |  | 2 | 9 |
| **Vaccine policy** | 5 | 1 |  | 1 | 7 |
| **Vaccine supply** | 3 | 1 |  | 1 | 5 |
| **Vaccine delivery** | 1 | 2 |  | 1 | 4 |
| **Civil society** | 1 | 0 |  | 0 | 1 |
| **Academic** | 3 | 1 |  | 2 | 6 |
| **Ministry of Health** | 2 | 0 |  | 4 | 6 |
| **Ministry of Finance** | 1 | 0 |  | 0 | 1 |
| **Other Government** | 0 | 4 |  | 0 | 4 |
| **NGO** | 1 | 1 |  | 2 | 4 |
| **Total** | **34** | **21** |  | **26** | 82 |

TB= Tuberculosis; NGO= non-governmental organization
