# Supplementary material for: Potential implementation strategies, acceptability, and feasibility of new and repurposed TB vaccines
Source: PLOS Glob Public Health. 2022 May 3;2(5):e0000076. doi: 10.1371/journal.pgph.0000076 (PMC10021736; doi:10.1371/journal.pgph.0000076)
Supplement: S1 File — (DOCX) [file pgph.0000076.s002.docx]

**S1 File. Background information.**

**Modelling of new TB vaccines suggests that greater and faster impact would be achieved by vaccinating adolescents/adults, instead of children, with new TB vaccines (Knight et al 2014). Therefore, we are trying to understand how new TB vaccines might be delivered to adolescents/adults and the associated costs of delivery.**

**For the M72/AS01E /AS01E vaccine (M72/AS01E),** a phase IIB study in South Africa, Zambia and Kenya suggested the vaccine would reduce the development of pulmonary tuberculosis disease by ~50% when given to HIV negative populations aged 18-50 years that were already-infected with *Mycobacterium tuberculosis* (as determined by IGRA-positivity) at time of vaccination. The vaccine was effective for up to 3 years.

The vaccine is known to be safe and induce an immune response in IGRA-negative populations and HIV-positive populations, but its efficacy in these populations is currently unknown as it has not been explored in clinical trials.   Again, modelling suggests that greater and faster impact would be achieved if vaccinating adolescents/adults instead of children with new *TB vaccines.*

**For revaccination with the BCG vaccin**e, a phase IIB study in South Africa suggested that, when given to HIV negative adolescents aged 12-18 that are **NOT** infected with *Mycobacterium tuberculosis* (as determined by IGRA-negativity) at time of vaccination, the vaccine reduced the development of sustained IGRA conversion (i.e., sustained *M. tb* infection) by ~45% (95% CI: 6.4 to 68.1). The vaccine is known to be safe when given to IGRA-positive (i.e., infected) populations, but its efficacy in these populations is unknown (though other studies have suggested potentially low efficacy if infected at time of vaccination). The vaccine is currently contraindicated in HIV-positive populations. Duration of protection of adolescent vaccination has not been studied, but infant vaccination has demonstrated duration of efficacy of at least 15 years, with some studies suggesting even longer durations are possible.
