# Supplementary material for: Potential implementation strategies, acceptability, and feasibility of new and repurposed TB vaccines
Source: PLOS Glob Public Health. 2022 May 3;2(5):e0000076. doi: 10.1371/journal.pgph.0000076 (PMC10021736; doi:10.1371/journal.pgph.0000076)
Supplement: S2 File — (DOCX) [file pgph.0000076.s003.docx]

# **S2 File. Interview guide.**

In-depth interview guide

Thank you for agreeing to participate in our study. I appreciate you taking the time to speak with me.

We are conducting these interviews to better understand the potential future implementation strategies and their associated costs for adult/adolescent vaccination with possible future TB vaccines. We also hope to better understand the potential challenges (e.g., political, financial, logistical) associated with the implementation of new TB vaccines for adolescents and adults.

We will talk for about an hour to an hour and a half, but you can stop at any time. You do not need to answer any questions that you don’t want to answer. It is fine to answer only what you are comfortable talking about. There is no right or wrong answer to these questions. Your perspective really matters to us, so we really want to hear what you think and what your experiences have been. Should we run out of time, would you be willing to schedule a follow up call? And if I have any further questions, can I contact you again?

Before we begin, I would like to gather some basic background information that is helpful to us:

| Site name/ID |  |
| --- | --- |
| Interviewer |  |
| Participant Code | *1* |
| Date of interview |  |
| Time of interview | Time started: |
|  | Time ended: |
| Which of the following are your areas of experience? (tick all that are relevant) | Tuberculosis        Vaccine development        Vaccine procurement/supply        Vaccine delivery        Health/programme financing        Regional/local level        National level        Other (please specify): __________________ |

Section 1: Semi-structured questions

**The purpose of this section is to explore/understand the acceptability of potential new TB vaccines, some of the potential challenges/barriers to introducing new TB vaccines for adolescents and/or adults, and to identify possible solutions. For interviewees in programme financing there is also discussion around prioritisation of financing.**

*Acceptability*

1. If an efficacious vaccine were available, do you think there would be interest in a new TB vaccine at the national/regional/individual level?
   1. And would there be any differences between the M72/AS01E and BCG revaccination?
2. If there were 3-4 pieces of data you consider critical to your country/program’s ability to make decisions on TB vaccine introduction, what would those be? (*E.g., Efficacy in specific high-risk group, clinical studies in local context; cost of delivery to adolescents, impact on TB burden if given to different target groups etc.)*

*Challenges/barriers/solutions*

1. Are there any aspects with regards to **possible characteristics of the vaccine** that you think are particularly important or could be problematic? Are there any aspects that could be a deal breaker?
   1. What do you think could be done to help overcome these?
2. Are there any potential **political** barriers to introducing an adult/adolescent TB vaccine?
   1. What do you think could be done to help overcome these?
3. Are there any potential **financial** barriers to introducing an adult/adolescent TB vaccine?
   1. What do you think could be done to help overcome these?
4. Are there any potential **logistical** barriers to introducing an adult/adolescent TB vaccine?
   1. What do you think could be done to help overcome these?
5. Are there any potential **acceptability barriers** at the individual or community level to introducing an adult/adolescent TB vaccine?
   1. What do you think could be done to help overcome these?
6. What challenges might be faced in introducing **routine** vaccination?
   1. What do you think could be done to help overcome these?
7. What challenges might be faced in introducing **mass** vaccination?
8. What do you think could be done to help overcome these?
9. Are there any challenges or barriers associated with **adding a vaccine to the existing immunisation programme**? (e.g., capacity, resources)
   1. What do you think could be done to help overcome these?
10. Are there any **other** challenges or barriers to introducing an adult/adolescent vaccine that you think might be important?
    1. What do you think could be done to help overcome these?
11. If those changes could be put into place, what impact do you think they would have on the introduction of the vaccine (e.g., more likely to be introduced, better coverage, better acceptance at the national/community/individual level)?

Section 2: Research questions

***We will continue with structured questions on the possible delivery of an adult/adolescent TB vaccines.* *Modelling of new TB vaccines suggests that greater and faster impact would be achieved by vaccinating adolescents/adults, instead of children, with new TB vaccines (Knight et al 2014). Therefore, we are trying to understand how new TB vaccines might be delivered to adolescents/adults and the associated costs of delivery. We will hereby focus on the M72/AS01E candidate and BCG revaccination***

***Did you have any questions about the background information we send about the M72/AS01E candidate and BCG revaccination***

1. For the M72/AS01E /AS01E vaccine:
2. Which populations would you vaccinate (e.g., age groups, risk groups, everyone?)
3. What do you anticipate the rough population size is? (1000s, 10 000s, 100 000s)
4. Would you use routine vaccination (at a given time or age), or mass campaigns to vaccinate this group
5. How and where would the vaccine be delivered as described above? (e.g. at existing interaction with the health service, school, work place, community campaigns)
6. Which of these groups would be prioritized in roll-out?

For the target groups identified in question 1a-e: focus on all-pop, age-related, HIV status and SES, focus biggest population, high priority risk group:

1. How much time after registration / policy recommendation expect to start
2. What coverage do you think would be achieved (for routine maintained coverage, for mass peak coverage)?
3. How quickly would that coverage be achieved?
4. For mass, what would be the frequency and length of campaigns (years)
5. How much do you think it would be reasonable to spend per year on a TB vaccination program like this? How have you come to this amount?
6. What is the % of GDP
7. From which budget does this come
8. Regarding M72/AS01E : If the vaccine is effective for 10 years, how would that change any of your answers?
9. For revaccination with the BCG vaccine:
10. Which populations would you vaccinate (e.g., age groups, risk groups, everyone?)
11. What do you anticipate the rough population size is? (1000s, 10 000s, 100 000s)
12. Would you use routine vaccination (at a given time or age), or mass campaigns to vaccinate this group
13. How and where would the vaccine be delivered as described above? (e.g. at existing interaction with the health service, school, work place, community campaigns)
14. Which of these groups would be prioritized in roll-out?

For the target groups identified in question 1a-e: focus on all-pop, age-related, HIV status and SES, focus biggest population, high priority risk group:

1. How much time after registration / policy recommendation expect to start
2. What coverage do you think would be achieved (for routine maintained coverage, for mass peak coverage)?
3. How quickly would that coverage be achieved?
4. For mass, what would be the frequency and length of campaigns (years)
5. How much do you think it would be reasonable to spend per year on a TB vaccination program like this? How have you come to this amount?
6. What is the % of GDP
7. From which budget does this come
8. Regarding BCG revaccination: If this will be found effective for Mtb infected (IGRA+) individuals, how would that change your answers?

**For the following questions, if the question is outside of the area of expertise/knowledge of the interviewee, note this down and skip to the next question.**

*Tuberculosis/HIV control programmes (only for participants answering yes to tuberculosis experience)*

1. Do you think there will be changes in the TB control programme in the future (e.g. changes to treatment regimens or other control measures)? If so, what do you think will change, how do you think it will change and when?
2. Do you think there will be changes in the HIV control programme in the future (e.g. changes to treatment regimens or other control measures)? If so, what do you think will change, how do you think it will change and when?
3. How do you think coordination between TB and vaccine programs will work on policy, funding and execution?

*Experience with existing adult/adolescent vaccines*

1. Has a new vaccine for adolescents and/or adults been introduced in to the vaccination schedule in South Africa)? If yes, which vaccine, who was it given to, how was it delivered to that population, and what coverage was achieved?  If no, skip to Section 2-.
2. Can you describe some of the logistical details of how the vaccine was introduced and delivered? If no, skip to Q8. If yes, can you describe the costs of the resources and services you just described? If you do not know the cost, do you know where this data could be found?
3. Which budgets did these come from (i.e. who was the payer for the different items)?
4. How much was/is spent per year on this vaccination program?
5. If the interviewee described the introduction of a vaccine for adolescents/adults in the previous questions: what were some of the challenges/barriers to introducing that vaccine? Would those same challenges be faced with the introduction of an adult/adolescent TB vaccine?

*Prioritisation of financing*

1. Are you able to share the approximate governmental budget for the TB programme, the immunisation programme, and/or the HIV programme? If you do not know the budget, do you know where this data could be found?
2. % GDP
3. Who would need to give approval for reallocation of existing funding or allocation of new funding to cover the resource requirements of this program? What do you think they would consider in making this decision?
4. When thinking about a potential budget for a TB vaccine you consider impacts on other areas beyond the direct costs and benefits of this program when thinking about how much to spend? *(For example:*

*● Reduced treatment costs of recipients in the future*

*● Impacts on other disease areas e.g. if HIV patient is prevented from dying from TB then have to treat patient for HIV for longer*

*● Impacts on other members of the family of the treated patient e.g. financial impoverishment, caring time and effort etc.*

*● Impacts on the wider economy in terms of the economic contribution of a healthy population*

*● Any other impacts)*

1. If economic impact estimations are available who would we best run those by?

*Regulatory process*

1. Would it be possible to seek a fast-track regulatory process for approval of the vaccine, once available? And if so would you seek this type of process?

Wrap-up

1. Is there anything you would like to add that you did not have a chance to say?

**Thank you very much for taking the time for this interview. Your contribution to this research is greatly appreciated, and it was very nice to meet you and discuss with you today. If you would like to get in touch about the study, the contact details for the study team are on the information sheet that you received.**
