# Supplementary material for: Potential implementation strategies, acceptability, and feasibility of new and repurposed TB vaccines
Source: PLOS Glob Public Health. 2022 May 3;2(5):e0000076. doi: 10.1371/journal.pgph.0000076 (PMC10021736; doi:10.1371/journal.pgph.0000076)
Supplement: S3 File — (DOCX) [file pgph.0000076.s004.docx]

**S3 File. Informed consent.**

**Informed Consent to Participate in Research**

**CONSENT**

I have been informed about the study: Semi-structured interviews to inform the translational development of late-stage tuberculosis vaccine candidates by Puck Pelzer.

I understand the purpose and procedures of the study.

I have been given an opportunity to ask questions about the study and have received answers to my satisfaction.

I declare that my participation in this study is entirely voluntary and that I may withdraw at any time without affecting my relationship with the research team.

I agree to the interview being audio recorded. I have been informed about any available compensation as a result of study-related procedures.

If I have any further questions/concerns or queries related to the study I understand that I may contact the researcher at:

Professor Janet Seeley, London School of Hygiene and Tropical Medicine, 15-17 Tavistock Place, London, UK

Phone: +44 (0)2079588268

E-mail: janet.seeley@lshtm.ac.uk

If I have any questions or concerns about my rights as a study participant, or if I am concerned about an aspect of the study or the researchers then I may contact the LSHTM Research Ethics Committee, contact details as follows:

Professor Jimmy Whitworth, Chair of London School Of Hygiene and Tropical Medicine Ethics Board, London School of Hygiene and Tropical Medicine, 15-17 Tavistock Place, London, UK

Tel: +44 (0)2079272418

Email: ethics@lshtm.ac.uk

____________________ ____________________ _________

Name and surname of participant Signature of participant Date

____________________ _____________________ _________

Name and surname of interviewer Signature of interviewer Date
